# 1130. Impact of a New Pediatric Antibiotic Stewardship Program on Ceftriaxone Use at an Academic Medical Center

**DOI:** 10.1093/ofid/ofab466.1323

**Published:** 2021-12-04

**Authors:** Derek Evans, Mariana M Lanata Piazzon, Kaitlyn Schomburg

**Affiliations:** 1 Cabell Huntington Hospital, Huntington, West Virginia; 2 Marshall University, Huntington, West Virginia

## Abstract

**Background:**

Hoop’s Family Children’s Hospital is a pediatric hospital with 72 beds, nested within Cabell Huntington Hospital. There is an established adult antibiotic stewardship program (ASP), however, since 2014 there has not been a pediatric infectious disease (ID) specialist and no pediatric ASP. With the recent hire of a pediatric ID specialist in Oct 2019 and the formation of a targeted pediatric ASP, we tracked the use of ceftriaxone (CRO) in our facility.

**Methods:**

Starting January 2020, education was provided to pediatric providers in regards to appropriate CRO dosing and clinical indications via email communication. The main goals were to limit 100mg/kg/day dosing to severe infections and reduce CRO use in community-acquired pneumonia. This was sustained through intermittent prospective audits and feedback. A retrospective chart review was done from 2019-2021 for the months of January, April and December of each year. Patients ≤18 years of age who received CRO were included. Dosing, interval frequency, indication, and treatment duration were reviewed. Patients who received a single dose of CRO were excluded.

**Results:**

From Jan 2019 – April 2021, 391 patient charts were reviewed (189 in the pre-intervention period and 202 in the post intervention period). There were no significant differences in age, race/ethnicity and gender in the two study groups. In the pre-intervention period, 86% of patients were prescribed CRO at severe infection dosing vs 33% in the post intervention period (p< 0.0001) (Figure 1). When dosing was paired with indication, only 20% of patients in the pre intervention period had the appropriate dosing per clinical indication compared to 83% in the post intervention period (p< 0.0001) (Figure 2). We also saw that in the pre-intervention period the most common indication for CRO was pneumonia (66%), which decreased to 57% in 2020 and to 35% in 2021 (p< 0.0001) (Figure 3).

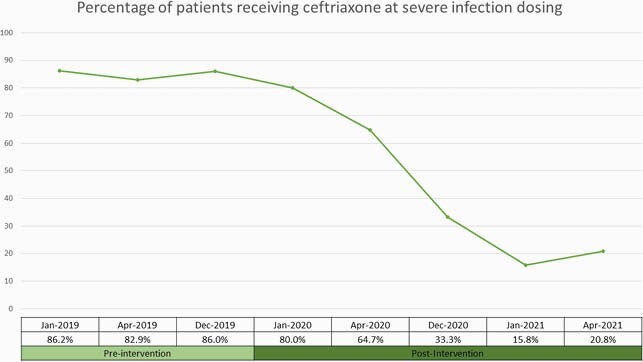

Figure 1 describes the percentage of patients receiving ceftriaxone at severe infection dosing. This changed from an average of 86% in the pre-intervention period to 33% in the post-intervention period.

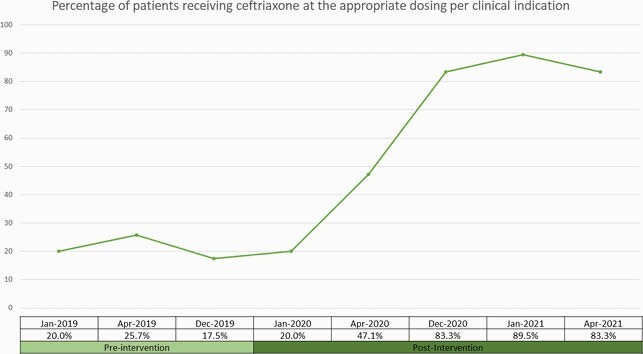

Figure 2 describes the percentage of patients receiving ceftriaxone at the appropriate dosing dependent on the clinical indication provided. This changed from 20% in the pre-intervention period to closer to 90% in the post-intervention period.

**Conclusion:**

Pediatric specific ASP efforts and expertise proved to be crucial in appropriate CRO use in our institution. With a feasible education strategy and targeted prospective audit and feedback, there has been a sustained impact in inappropriate CRO use. This underscores the importance of targeted pediatric ASP efforts in pediatric hospitals within larger adult hospitals.

**Disclosures:**

**All Authors**: No reported disclosures

